# Biomechanical Performance of BoneHelix^®^ Compared with Elastic Stable Intramedullary Nailing (ESIN) in a Pediatric Tibia Fracture Model

**DOI:** 10.3390/life11111189

**Published:** 2021-11-05

**Authors:** Laura Leonie Brandes, Luis Fernando Nicolini, Johannes Greven, Philipp Lichte, Thomas Thaddäus Stopinski, Martin Sattler, Frank Hildebrand, Miguel Pishnamaz

**Affiliations:** 1 Department of Orthopedics, Trauma and Reconstructive Surgery, RWTH Aachen University Hospital, Pauwelsstr. 30, 52074 Aachen, Germany; laura.brandes@rwth-aachen.de (L.L.B.); lnicolini@ukaachen.de (L.F.N.); jgreven@ukaachen.de (J.G.); plichte@ukaachen.de (P.L.); fhildebrand@ukaachen.de (F.H.); 2 Institut für Versuchstierkunde, RWTH Aachen University Hospital, Pauwelsstr. 30, 52074 Aachen, Germany; tstopinski@ukaachen.de; 3 Johannes Wesling Klinikum Minden, Hans-Nolte-Straße 1, 32429 Minden, Germany; Martin.Sattler@muehlenkreiskliniken.de

**Keywords:** fracture, tibia, pediatric, cyclic testing, elastic stable intramedullary nailing (ESIN), BoneHelix^®^, biomechanics

## Abstract

Tibial shaft fractures are common injuries in the pediatric and adolescent populations. Elastic stable intramedullary nailing (ESIN) is the treatment of choice for cases that require surgical stabilization. A new intramedullary device, BoneHelix^®^ (BH), may be an alternative for use with fractures that cannot be satisfactorily stabilized with ESIN. This study aimed to assess the biomechanical performance of BH compared with ESIN in a porcine tibia fracture model, observing cyclic fatigue and load to failure. Computed tomography was used to monitor the implant position and to rule out unintended damage. No implant or bone failure occurred during the fatigue testing. An increase in the cumulative plastic displacement was observed in both test groups over the loading cycles applied. Both implant–bone constructs displayed a trend toward closure of the osteotomy gap. During the load-to-failure test, the average loads at failure in specimens instrumented with ESIN and BH were 5364 N (±723) and 4350 N (±893), respectively, which were not statistically significant (*p* = 0.11). The values of both groups were two to three times higher than the estimated maximal load (2000 N) during physiological weight bearing. The biomechanical results thus indicate equivalent performance and stability by the implants tested.

## 1. Introduction

Tibial shaft fractures are among the most common skeletal traumas seen in children and adolescents [1]. Isolated tibial fractures with spiral or oblique fracture lines are the most frequent type [2]. The choice of treatment is based on several factors, including age, fracture pattern, associated soft-tissue injuries, and comorbidities [3,4]. Many cases can be managed satisfactorily with nonoperative treatment by closed reduction and casting [3,5,6,7]. These are usually associated with fast healing and a low complication rate [4,8]. However, surgical treatment is required in fractures that cannot be aligned and secured within the limits of spontaneous correction by using conservative methods [6,7,9]. Elastic stable intramedullary nailing (ESIN) is currently considered the gold standard for surgical fracture management.

Since it was first introduced in the early 1980s, ESIN has established itself as the surgical treatment of choice [10,11,12]. It is based on a minimally invasive approach using two titanium or stainless steel elastic nails to form a double arch inside the medullary cavity, providing a three-point-fixation [11,13,14]. Micromovements at the fracture zone are possible due to the flexible character of the nails [15], resulting in secondary bone healing. Nevertheless, ESIN does not come without its flaws. Possible complications include delayed union or malunion, failed fixation, leg length discrepancy, and superficial infection, among others [16,17,18,19,20,21,22,23]. Tibial fracture fixation with ESIN can be demanding, and the achievement of a correct three-point fixation is not always easy to assess intraoperatively.

BoneHelix^®^ (BH) is a helical steel coil spring with limited bending flexibility, designed for the intramedullary stabilization of long bone fractures [24,25,26]. Its multiple coils provide long-segmental contact to the spongiosa and corticalis of the bone, generating internal friction, while a high vertical spring rate prohibits impression and secures axial stability [27]. Its strictly limited flexibility allows micromovements at the fracture zone, which encourage callus formation, while preventing excessive bending [24]. In the pediatric tibia, the helix is inserted in the antegrade direction, sparing the open physis. Fixation of the implant is achieved by traction. Exercise and partial loading with a walker are allowed immediately after surgery [28]. A clinical study shows promising results in adult humeral shaft fractures treated with BH [29].

The present experimental study aimed to investigate and compare the biomechanical performance of BH and ESIN in cyclic fatigue testing, simulating an 18-week postoperative period [30], and also in load to failure, using a pediatric tibial fracture model.

## 2. Materials and Methods

### 2.1. Sample Acquisition and Preparation

Experiments were performed on eight pairs of fresh frozen tibiae, collected from juvenile pigs with an average weight of between 30 and 40 kg (Department for Laboratory Animal Science, RWTH Aachen University Hospital). Due to close similarities in anatomy and morphology, porcine bone is a suitable alternative to human bone for investigation of bone implants [31,32]. Specimens were stored at −18 °C and were thawed for over 16 h at 8 °C before testing [33,34]. Each tibia was separated from the knee joint and the fibula, and all soft tissue was removed from the bone. The tibiae were then fixated at their distal end in a 7° valgus alignment in a methyl methacrylate (MMA)-based synthetic material (Technovit^®^ 3040, Kulzer GmbH, Hanau, Germany). The valgus alignment represents the physiological leg axis in adults [30] and is completed in children by the age of 8 to 10 years [35,36]. The fixation was performed with the help of a custom-made rail system, a stencil, and a laser to achieve the correct alignment.

### 2.2. Surgical Groups and Implantation Procedure

Each pair of bones was randomly instrumented with a gliding nail system for ESIN (Königsee Implantate GmbH, Allendorf, Germany) or a BH device (H&R Medizintechnik GmbH & Co. KG, Lennestadt, Germany), in either the left or the right tibia.

**Figure 1:**
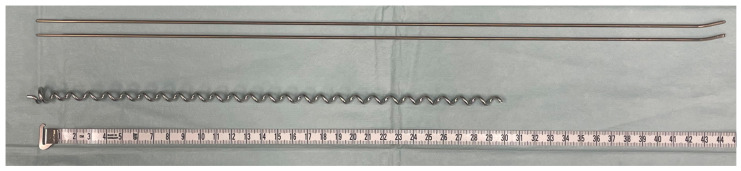
Implants compared in biomechanical testing: titanium gliding nails for ESIN with 2.0 mm diameter (above) and BH C1 implant (below); measuring tape is displayed in cm.

For ESIN, titanium nails with wire diameters of 2.0 and 2.5 mm and a length of 450 mm with flattened ends were selected in accordance with the bone size and the expected diameter of the intramedullary canal (Figure 1). The nails were implanted to fill 80% of the intramedullary canal isthmus [9,13].

BH implants are made from surgical long-term implant steel alloy (1.4441). The pediatric sizes C1 and C2, measuring an outer diameter of 7.0 or 8.0 mm, respectively, were used in accordance with the bone size (Figure 1). The aim is for the diameter of the selected helix to be 1 mm less than the narrowest point of the tibial diaphysis [28]. Implants intended for children have a standard length of 300 mm and a wire gauge of 2.5 mm.

For ESIN, two prebent C-shaped nails were introduced into the medullary cavity through openings medial and lateral to the tibial tuberosity using an inserter. The aim was to position the bent nails in a double-arch configuration within the medullary cavity. Their final position was secured by applying hammer taps to the pliers holding the nails.

The chosen BH was inserted into the medullary cavity via a guiding rod through a hole cranial to the tibial tuberosity. The helix was turned further in until resistance was noted, indicating the correct position inside the distal metaphysis. The construct was considered stable when at least six helical coils were positioned inside the bone.

**Figure 2:**
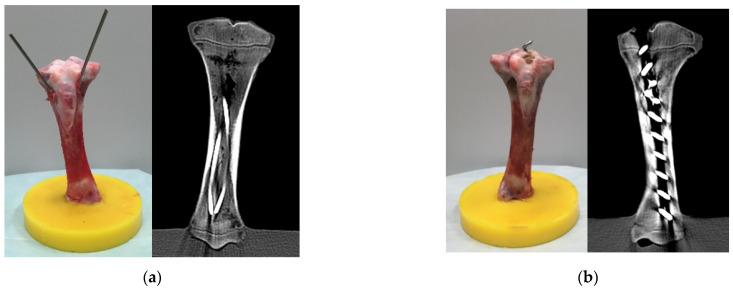
Specimens with ESIN (**a**) or BH (**b**) after instrumentation before fracture creation. The CT scans are shown on the right side of each subfigure.

The positions of both implants were checked with computed tomography (CT) (Figure 2).

The implants were trimmed to the minimum, and the proximal ends of the specimens were embedded. It was ensured that the implant ends had no contact with the embedding material so that the compressive load was transferred to the implant via the bone, and no force was acting directly on the implants during testing under compressive loads. A transverse midshaft fracture (classified as a 42t-D/4.1 fracture by PCCF [37] or a 4.2.s.3.0 fracture by LiLa [38]) was created 7 cm from the distal epiphysis using a handheld saw. A gap of 3 mm was kept between the fracture fragments to establish an unstable situation [39].

### 2.3. Biomechanical Tests

**Figure 3:**
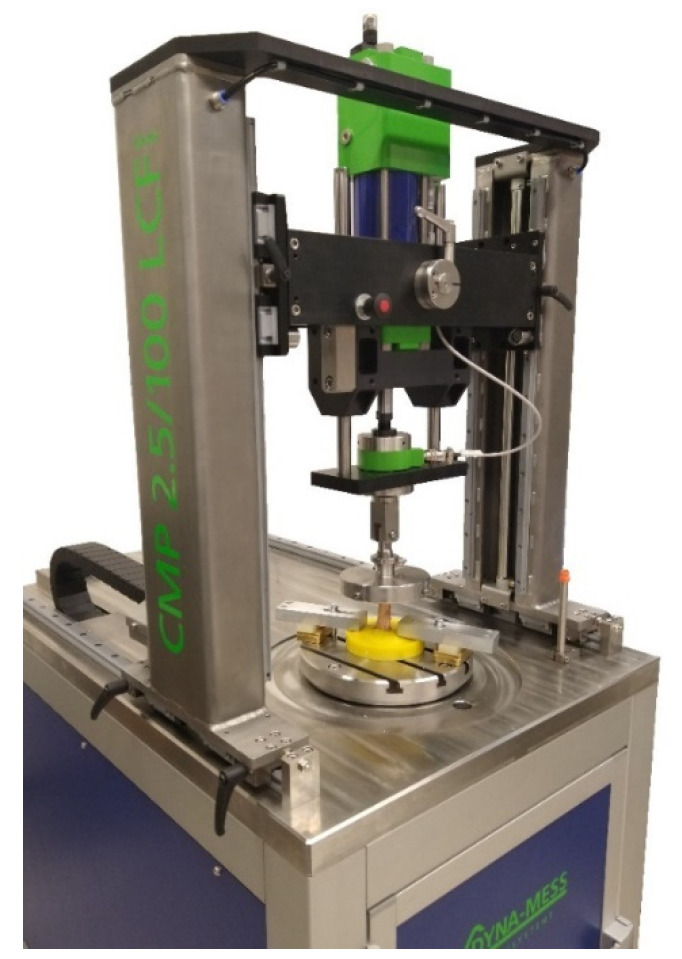
DYNA-MESS machine used for fatigue tests of the specimens. The specimen is a porcine bone used for pilot tests.

**Figure 4:**
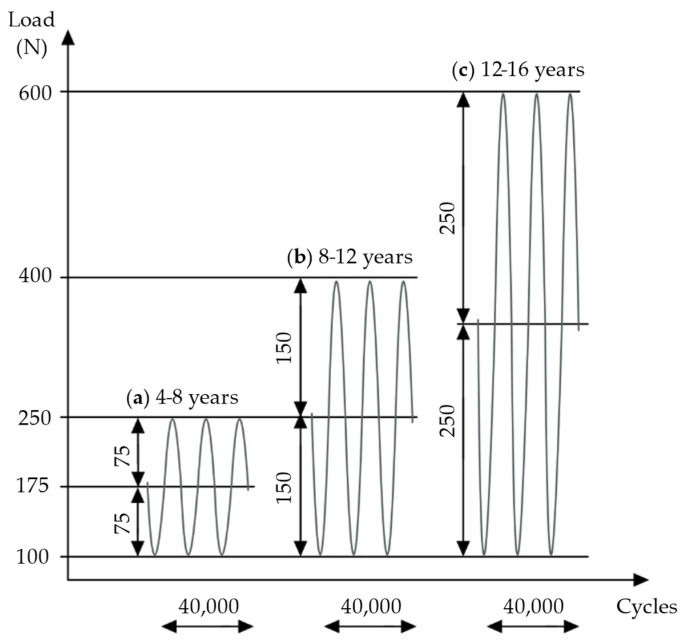
Three sequences of cyclic loading were applied: (**a**) load according to weight at 4–8 years, (**b**) load according to weight at 8–12 years, (**c**) load according to weight at 12–16 years. Own figure, based on Tschegg et al. [30].

All biomechanical tests were performed at room temperature. A servo-pneumatic material testing machine (DYNA-MESS Prüfsysteme GmbH, Stolberg, Germany) was used for the cyclic compressive fatigue testing (Figure 3). Each implant–bone construct was exposed to three series of increased sinusoidal cyclic loading, each including 40,000 test cycles. In total, 120,000 load cycles at a frequency of 2 Hz were performed. The chosen load protocol (Figure 4) followed a protocol by Tschegg et al. [30], which was designed to simulate physiological loading conditions on the tibia.

One series of 40,000 cycles simulates normal weight bearing for a 6-week postoperative period [30]; therefore, with the present experimental design, about 18 weeks of physiological loading was simulated. Applied loads were chosen according to the average weights of children in various age groups [40]. In the first test series, the load oscillated from a middle compressive load of 175 N, with an amplitude of 75 N, simulating the full body weight (BW) of children aged 4–8 years. The second test series applied 250 N at middle load, with a 150 N amplitude, simulating full BW at 8–12 years. In the third series, loading was increased to 350 N at middle load, with an amplitude of 250 N, simulating full BW at 12–16 years (Figure 4).

**Figure 5:**
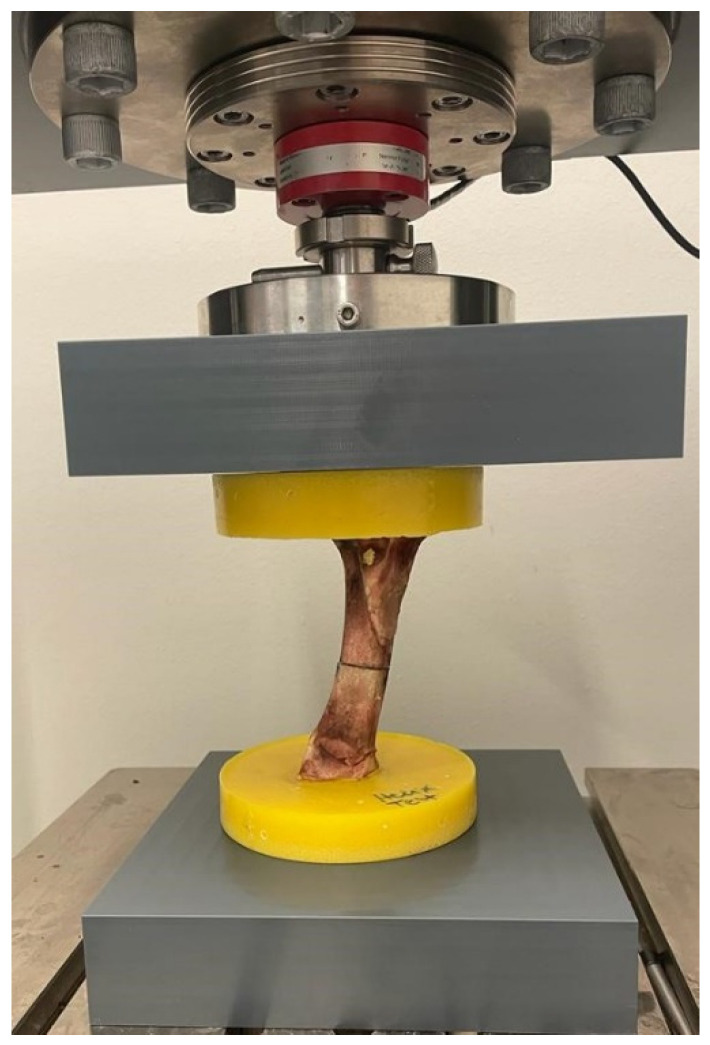
ZwickRoell machine used for load-to-failure tests. The specimen is a porcine bone used for pilot tests.

If 120,000 cycles of fatigue loading were tolerated without failure, the bone–implant construct was exposed to axial compression testing. A material testing machine (modernized with RetroLine testControl II, ZwickRoell GmbH & Co. KG, Ulm, Germany) was used to determine the individual maximal load until failure occurred for each bone–implant construct (Figure 5). Tests were started with a preload of 100 N and a testing speed of 1 mm/s until a predefined upper force limit was reached, or until a reduction of 10% of the applied load was detected, implying specimen failure (force shutdown threshold at 10%). Upper force limits (Table 1) were chosen according to maximal peak forces expected on the tibia in a subject of 60 kg (approximately girls at 16 years and boys at 15 years [40]) during normal daily activities.

Peak forces reach 2 to 2.5 times the BW during level walking and 3.5 times the BW when descending stairs [41]. A maximal peak force of about 1200–1500 N for walking and of about 2000 N for descending stairs can be derived from this consideration. Load-to-failure tests started with an upper force limit of 1000 N (approximately 50% of expected peak load) and were continued with 1500 N (75%) and 2000 N (100%) if no signs of failure were observed. Loading continued in steps of 1000 N until failure in the form of visual fracturing, implant failure, or embedding failure occurred.

### 2.4. Data Acquisition and Analysis

**Figure 6:**
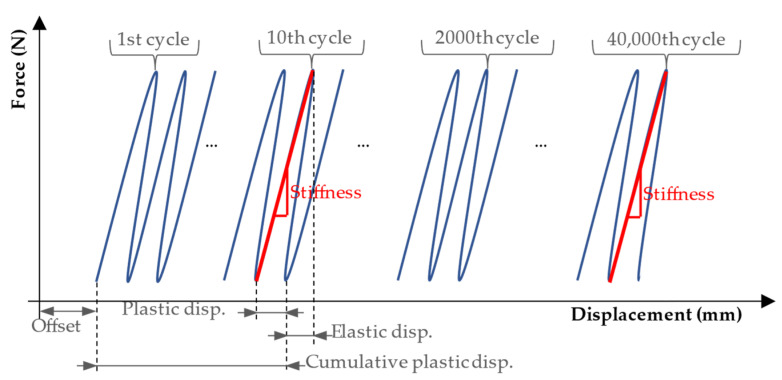
Definitions of elastic displacement and plastic displacement calculated for one cycle (loading and unloading), and cumulative plastic displacement calculated until the end of the 10th cycle. Stiffness was calculated by taking the slope considering the force–displacement datapoints at the maximum and minimum force (100 N) of the half cycle. Source: adapted from Tschegg et al. [30].

Load and axial displacements were recorded by a load cell and a displacement sensor at a frequency of 100 Hz in both fatigue and quasi-static compression tests. The sensors have a maximum error of 1% with respect to the target value. The measured axial displacements correspond to the reduction in specimen size. The first 10 cycles were used as preconditioning for adjusting the machine to the specimens. The definitions of elastic displacement (ED), plastic/permanent displacement (PD), cumulative plastic displacement, and stiffness are shown in Figure 6. ED describes reversible displacement that is reduced to zero after load removal [42]. PD is defined as displacement that persists after unloading the specimen [30], and it is associated with a reduction of the fracture site during testing. These variables were calculated by considering the displacement of the piston of the machine at the time of maximal and minimal loading of a loading cycle at the beginning (10th test cycle), at an early stage of testing (2000th test cycle), and at the end (40,000th test cycle) for each loading sequence (Figure 6). The ED was calculated by subtracting the PD from the total displacement. Stiffness was calculated by taking the slope considering the force–displacement datapoints at the maximum and minimum force. The cumulative plastic displacement represents the permanent displacement that occurred in the specimen from the beginning of the test until a certain cycle.

The fracture gap size was additionally measured with a digital caliper before and after cyclic testing. The caliper had an accuracy of ±0.2 mm and a resolution of 0.1 mm. CT images were captured by a 2 × 128-slice SOMATOM Definition Flash CT scanner (Siemens Healthcare AG, Zurich, Switzerland). Specimens were scanned after implant instrumentation to confirm the correct position inside the bone, after the cyclic fatigue tests, and again after the quasi-static compression tests were completed. Images were viewed and analyzed with the software IntelliSpace PACS Enterprise (version 4.4, Philips GmbH Market DACH Health System, Hamburg, Germany).

Statistical analyses were performed in Microsoft Excel (version 16.44, Microsoft Corporation, Redmond, DC, USA). The means of both test groups were compared using two-sample two-tailed *t*-tests or paired *t*-tests, respectively, when looking at values within the same test groups at different times of testing. All statistical tests were based on a 5% alpha error. Results with *p* values < 0.05 were considered statistically significant.

For the load-to-failure tests, the shortening of the specimen was analyzed from a load of 150 N to a maximum value representing a physiological situation (e.g., 1000 N). This load of 150 N served to reduce to zero the gap between the parts of the machine (e.g., interface of the specimen with the mechanical parts of the machine) so that the obtained displacement would be only from the specimen. The software G*Power (version 3.1.9.6) [43] was used to perform a power analysis at the point of discussing the maximal load tolerated at the point of failure.

## 3. Results

### 3.1. Cyclic Loading Fatigue Testing

All 16 specimens survived 120,000 cycles of increased fatigue loading without showing signs of implant or bone failure. This statement is supported by the data collected during testing and the CT imaging after the experiments were completed.

#### 3.1.1. Axial Displacements

**Figure 7:**
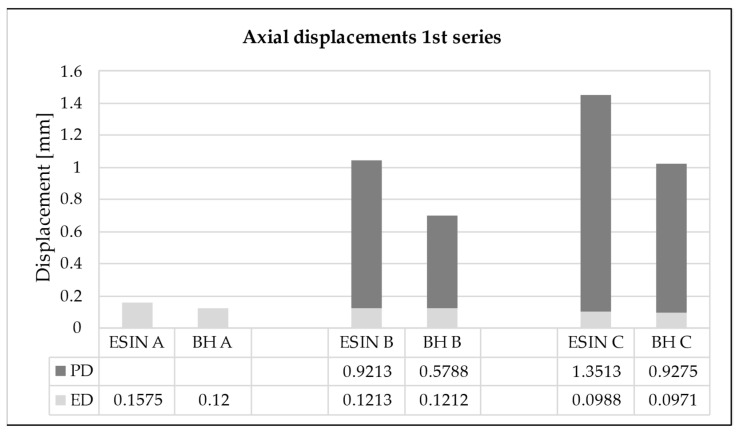
Elastic displacement (ED) and cumulative permanent displacement (PD) for the (A) beginning (10th test cycle), (B) early stage (2000th test cycle), and (C) end (40,000th test cycle) of the first loading sequence. ESIN specimens showed significantly greater cumulative PD than BH specimens (*t*-test; *p* = 0.014).

ED was significantly reduced in the ESIN specimens in all loading series between the 10th and 40,000th test cycle (paired *t*-tests: first: *p* = 0.0028, second: *p* = 0.0032, third: *p* = 0.0036). For the BH specimens, the reduction of ED was not verified to be statistically significant (paired *t*-tests: first: *p* = 0.058, second: *p* = 0.42, third: *p* = 0.39). Both implants showed a decreasing trend in the development of ED in all test series. However, the change in ED within each series was negligible (Figure 7). For the first loading series, it was 0.059 mm (±0.042) for ESIN and 0.023 mm (±0.032) for BH.

**Figure 8:**
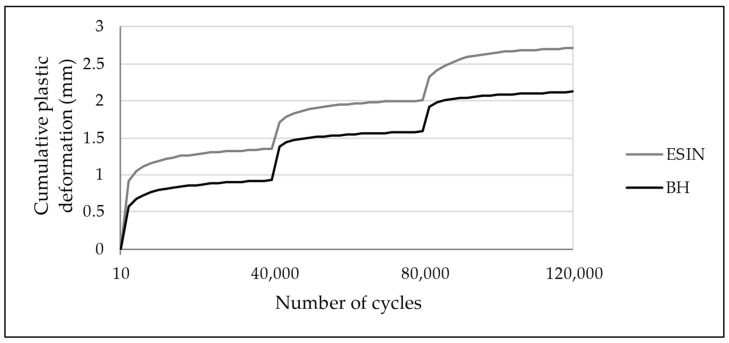
Development of cumulative PD over 120,000 test cycles with increased loading at 40,000th and 80,000th cycles. PD was greatest during the first 2000 cycles of each test series. ESIN specimens showed a significantly greater PD during the first loading sequence (*t*-test; *p* = 0.014). No statistically relevant difference in the development of cumulative PD was observed in the subsequent test series (*t*-test: second: *p* = 0.98, third: *p* = 0.13).

An increase in the cumulative PD was observed in both test groups over the loading cycles applied. The PD was greatest during the first 2000 loading cycles for each sequence when the force was increased, presenting an asymptotic behavior (Figure 8). For the first series, in the 2000th cycle it reached values of 0.92 and 0.58 mm for ESIN and BH, respectively (Figure 7). In the 40,000th cycle, the cumulative PD was 1.35 mm for ESIN and 0.93 mm for BH. The ESIN specimens showed a significantly greater cumulative PD than the BH specimens (*t*-test; *p* = 0.014).

Cumulative PDs of 2.71 mm (±0.71) in the ESIN and of 2.12 mm (±0.58) in the BH specimens were seen after 120,000 cycles of fatigue loading (*t*-test; *p* = 0.092). These results display an equivalent accumulated PD after simulated physiological weight-bearing modelling for an 18-week period. The manually measured reduction in the fracture gap (ESIN: 2.0 mm (±0.5), BH: 1.3 mm (±0.7)) showed the same trend toward closure, analogous to the increase in PD.

#### 3.1.2. Axial Stiffness

An increase in stiffness was observed for both test groups in relation to the increasing number of test cycles (Table 2). The increase was confirmed to be statistically significant for the ESIN group in all test sequences (paired *t*-test: first: *p* = 0.00016, second: *p* = 0.00028, third: *p* = 0.00081). However, the BH group only showed a significant increase in stiffness in the first and third sequences, but not in the second (first: *p* = 0.0088, second: *p* = 0.19, third: *p* = 0.0024).

Early stiffness was significantly higher in BH specimens in the second and third test series (*t*-test: second: *p* = 0.013, third: *p* = 0.026), while in the first series, no difference was revealed in early stiffness between groups (*p* = 0.91). This indicates a higher capacity for deformation, allowing more movement for specimens operating with ESIN under increased loading in comparison with BH. Late stiffness was equivalent for the test groups in all three test sequences (*t*-test: first: *p* = 0.89, second: *p* = 0.37, third: *p* = 0.57).

### 3.2. Load-to-Failure Testing

#### 3.2.1. Location and Type of Failure

**Figure 9:**
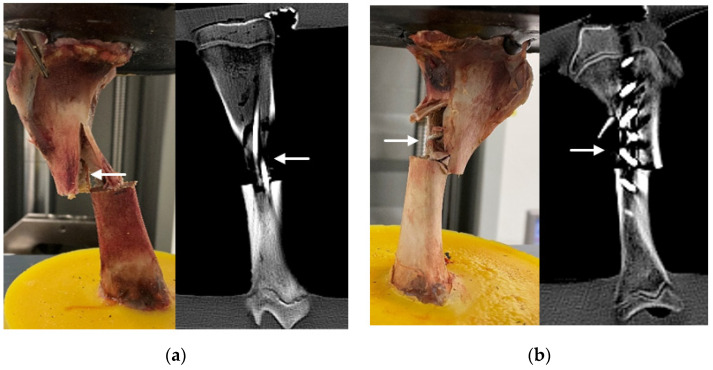
Implants after failure loading (examples): (**a**) ESIN specimen displaying a simple, displaced fracture after testing (left) and in CT (right); (**b**) BH specimen displaying multifragment, displaced fracture after testing (left) and in CT (right). Arrows indicate fractures. More examples are available as supplementary material in FigShare at DOI 10.6084/m9.figshare.16910611.

Neither of the test groups showed signs of implant failure during loading or in the following CT. A homologous fracture pattern was seen in both test groups. Fractures or fissures were located exclusively in the upper bone fragment, mostly in relation to the previously created transverse fracture site in the middle third of the tibia (Figure 9). An exception was seen in one ESIN specimen, where a fissure started at the lateral drill hole created for the implant insertion. Longitudinal stress fissures were displayed in three ESIN and two BH specimens. Incomplete, displaced, simple longitudinal fractures were seen in one ESIN and two BH specimens. In the BH group, fatigue loading resulted once in a complete, longitudinal, displaced multifragment fracture with blasted-out bone fragments.

#### 3.2.2. Load at Failure

Loadings of 50% (1000 N), 75% (1500 N), and 100% (2000 N) of the expected peak load were tolerated by all bone–implant constructs in both test groups. The mean maximal load detected at failure in both test groups was 4801 N (±885). This equals about 240% of the expected peak load of 2000 N. ESIN specimens tolerated 5364 N (±723) on average, while BH specimens were loaded with 4350 N (±893) before bone failure occurred (268% vs. 218% of the expected peak load). The maximal loads at failure did not differ significantly between test groups (*t*-test; *p* = 0.11). The original data for the load-to-failure tests are available as Supplementary Material in FigShare at DOI 10.6084/m9.figshare.16910611 (last accessed on 1 November 2021).

#### 3.2.3. Axial Displacement at Maximal Load

**Figure 10:**
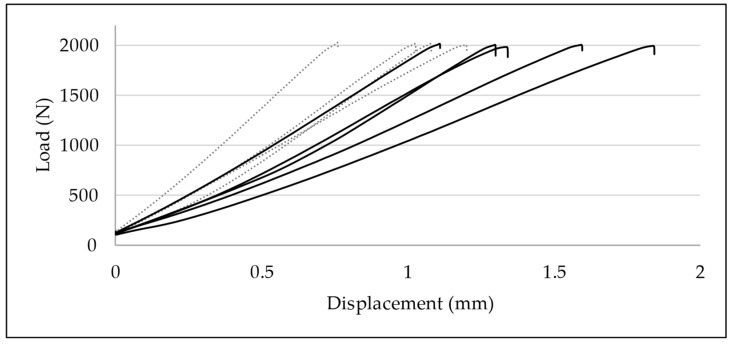
Load–displacement graphs of quasi-static compressive loading until 2000 N: development of axial displacement in ESIN (dotted lines) and BH specimens (continuous lines). Displacement was significantly greater in BH specimens (*p* = 0.037).

Load–displacement graphs display a linear, proportional, and statistically relevant increase in axial displacement according to increased loading in both test groups (Figure 10).

Displacement data at 1000, 1500, 2000, and 3000 N were compared for each group. In the ESIN specimens, displacement increased significantly from 0.55 mm (±0.18) at 1000 N to 0.75 mm (±0.14) at 1500 N (paired *t*-test; *p* = 0.025), to 0.99 mm (±0.18) at 2000 N (*p* = 0.001), and to 1.52 mm (±0.30) at 3000 N (*p* = 0.0034). In the BH specimens, displacement increased from 0.75 mm (±0.19) at 1000 N to 1.09 mm (±0.25) at 1500 N (*p* = 0.00020), to 1.40 mm (±0.27) at 2000 N (*p* = 0.000028), and to 2.32 mm (±0.57) at 3000 N (*p* = 0.0035).

No relevant difference of displacements between test groups was observed for the upper force limits of 1000 N (*t*-test; *p* = 0.17) and 1500 N (*p* = 0.050). In contrast, BH specimens displayed a greater axial displacement when loaded until 2000 N (*p* = 0.037) and 3000 N (*p* = 0.041) (Figure 10). BH specimens apparently suffered greater displacement than ESIN specimens when exposed to greater loading.

## 4. Discussion

This is the first study that compares the biomechanical effects of fracture stabilization by BH or ESIN on a porcine tibial fracture model simulating a pediatric trauma with 18 weeks of postsurgical mobilization. BH is an innovative treatment option for the treatment of long bone fractures, which are common skeletal trauma patterns. A clinical study on adult humeral shaft fractures treated with BH presented promising results [29]. However, limited data are available regarding the fatigue and load-to-failure performance of BH, which could contribute to clinical findings, and there is no analysis from the biomechanical point of view of the possibility of using BH as an alternative to ESIN.

Several studies attest that ESIN has a low complication rate and positive outcomes after treatment, and they promote it as an effective and reliable method for the surgical management of pediatric tibial shaft fractures [21,44,45,46,47].

However, possible complications, such as delayed union, malunion, and failed fixation, can occur [16,17,18,19,20,21,22,23]. The main reasons for the failure of ESIN are errors during the insertion process or, in particular, insufficient intraosseous three-point fixation. This problem can occur because of complex fracture scenarios (oblique or multifragmentary) or due to adverse angles of the insertion axis of ESIN in the long bone, resulting in a weak bracing of the wires in the bone shaft [13,20,48,49,50,51,52]. The rate of complications, such as malunion or delayed union, is also higher in heavier adolescent patients [9,16,17,18,19], although greater weight alone does not seem to predict a higher rate of malunion [16,46,53]. These limitations indicate the need to develop alternative surgical treatment options for cases in which ESIN does not provide sufficient stabilization for adequate bone healing.

Stabilization of the fracture is an important requirement for tibial implants. The implants need to withstand forces occurring at the knee joint under normal weight bearing during activities of daily life, since they must compensate for the temporary loss of function of the traumatized bone. The absence of signs of implant or bone failure as analyzed by visual inspection or with CT scans indicates that simulated physiological loading after surgical treatment of a tibial shaft fracture with ESIN or BH in the pediatric and adolescent age group is tolerated without causing failure of implants or of the osteosynthesis–bone construct.

This demonstrates that both types of implants are capable of fixing and keeping the bone parts together while the bone–implant construct is submitted to physiological loads adapted to the bone healing process of a child [54].

The increase in cumulative PD and the reduction of fracture gap present in both groups indicate that the load exceeded the yield strength of the bone–implant construct, causing it to compress. This effect led to an increase in the axial stiffness of the system when the results of the last series were compared against those of the first series. The cumulative PD tended to converge to a specific value until the external load was increased in our testing protocol, suggesting that the amount of damage tends to stabilize.

The results demonstrated that the bone–implant constructs tested can withstand loading beyond physiological conditions with no statistical differences between groups, while the magnitude of the load is limited to the weight tolerance of the fractured tibial bone structure.

For both implants, the load at failure was two to three times higher than the expected peak load experienced by the tibia of a subject of 60 kg performing daily activities, such as walking and descending stairs. Additionally, collapse of the bone or the interface with the embedding material was observed during load to failure; however, there were no signs of implant damage. This suggests that the weakest structure is the bone volume close to the fracture site, and therefore, from a purely mechanical point of view, further improvement of the instrumentation to resist higher forces would require a reduction of the forces exerted on the tibia close to the fracture site. The implant itself does not seem to be the problem in resisting physiological stress inside the tibia since none of the implants failed. Moreover, the bone instrumented with BH had an equivalent load tolerance to that instrumented with ESIN.

This study has some limitations. The in vitro testing differs from the in vivo condition due to the missing vasculature and soft tissue envelope, and also in terms of the loading condition. This was also the reason why no tests were performed with regard to the rotational stability of the two implant types, as the surrounding soft tissue plays a decisive role here. More complex tests must be carried out to simulate further physiological parameters and loads and to investigate whether or not BH is superior to ESIN in certain scenarios. The physiological forces acting on the tibia adapt to a complex system of muscular forces that change to control movements and to keep the body in equilibrium when external loads, such as the gravity, are acting. We approximated this configuration by using a compressive force that acted along the tibia shaft. However, several authors perform simplification of the loads [30,55,56,57,58], and this is frequently necessary to make the testing reliable. Furthermore, we used pig bones instead of human cadaver specimens. However, animal specimens do represent a suitable alternative to human bones because of their low variance in material properties, low cost, and availability [32].

In summary, all test results implied equivalent biomechanical performance by the two types of implants investigated, with BH providing higher stability in the early weight loading period, paired with greater stiffness than ESIN. This might be an advantage in the stabilization of more complicated fractures or in heavier adolescent patients in whom fractures need a longer time to form callus and are prone to develop complications [16,17,18,19]. Our model, specifically designed to address the issue of axial load parameters, provides the basis for further investigation of BH in regard to rotational stability. Nevertheless, for the first time we obtained results for axial load experiments to attest to BH’s satisfactory biomechanical properties for use in pediatric and adolescent tibial shaft fractures, which could compensate for the possible limitations of ESIN.

## Figures and Tables

**Table 1:** Load protocol of load-to-failure testing. First, peak loads of 50%, 75%, and 100% were applied. Loads were increased in steps of 1000 N until failure was observed. An amount of 2000 N was expected to be the tibial peak load in a subject of 60 kg, representing 3.5 times the body weight when descending stairs. A 100% peak load was estimated to cover the forces occurring in everyday activities.

Test	Upper Force Limit (N)	Percentage of Expected Peak Load
1	1000	50%
2	1500 (walking)	75%
3	2000 (stairs)	100% (3.5 × 60 kg)
4–*n*	3000–*n*	150%–*n*

**Table 2:** Development of axial stiffness in cyclic loading fatigue testing. A significant increase in stiffness was observed in ESIN specimens for all test series. Increase in stiffness in BH specimens was verified to be significant in the first and third test series only.

Series	Cycles	Stiffness ESIN (N/mm)	Stiffness BH (N/mm)
1	10 40,000	1044 (±606) 1685 (±733)	1070 (±191) 1639 (±513)
2	40,010 80,000	911 (±279) 1406 (±336)	1433 (±434) 1562 (±339)
3	80,010 120,000	1122 (±246) 1709 (±339)	1594 (±477) 1824 (±435)

## Data Availability

The data are available online in FigShare at DOI 10.6084/m9.figshare.16910611 (last accessed on 1 November 2021).

## Supplementary Materials

The following are available online in https://www.mdpi.com/article/10.3390/life11111189/s1 and FigShare at DOI 10.6084/m9.figshare.16910611 (last accessed on 1 November 2021). Figure S1: Images of specimens with ESIN implants after load to failure tests. Arrows indicate fractures; Figure S2: Images of specimens with BoneHelix implants after load to failure tests. Arrows indicate fractures; Table S1: Experimental data after load-to-failure tests of BoneHelix; Table S2: Experimental data after load-to-failure tests of ESIN.
