# Supplementary material for: Biomechanical Performance of BoneHelix® Compared with Elastic Stable Intramedullary Nailing (ESIN) in a Pediatric Tibia Fracture Model
Source: Life (Basel). 2021 Nov 5;11(11):1189. doi: 10.3390/life11111189 (PMC8622329; doi:10.3390/life11111189)
Supplement: Supplementary file 1 [file life-11-01189-s001.zip › Supplementary material - Pictures after load-to-failure tests.pdf]

life

MDPI

*Supplementary material*

Examples of fractures obtained after load-to-failure tests with ESIN and BoneHelix are shown in Figure S1 and Figure S2, respectively.

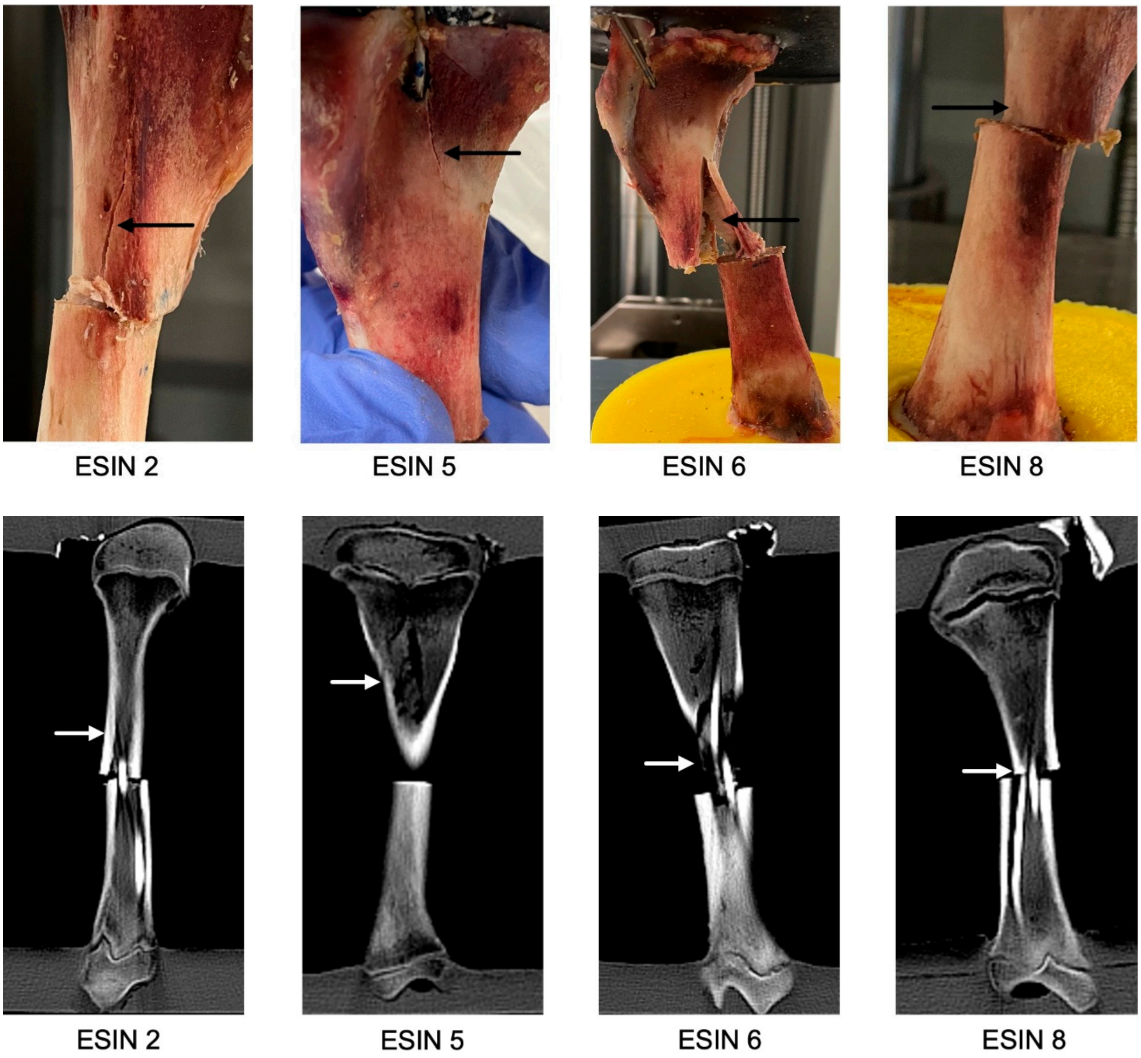


**Figure S1.** Images of specimens with ESIN implants after load to failure tests. Arrows indicate fractures.

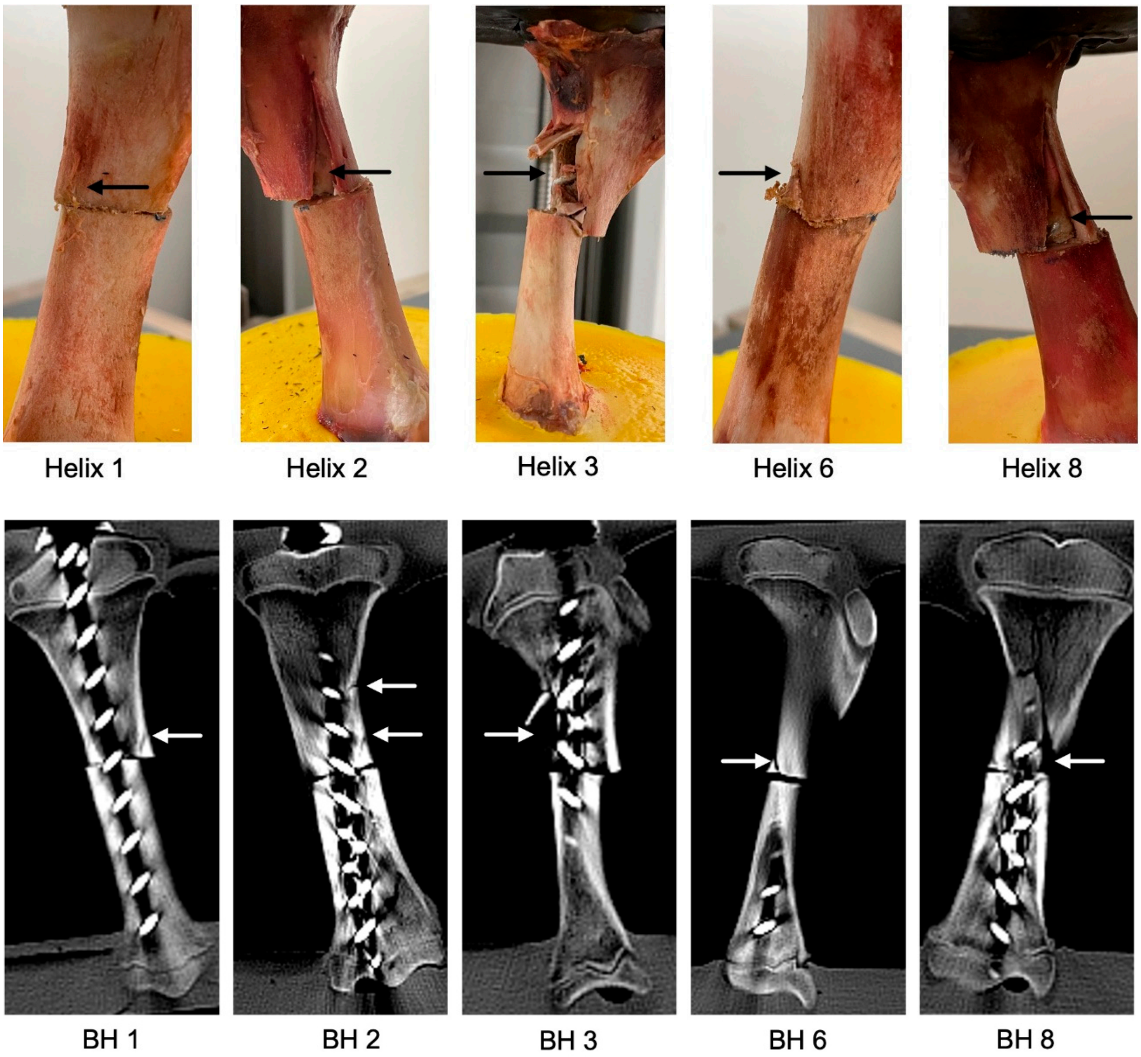


**Figure S2.** Images of specimens with BoneHelix implants after load to failure tests. Arrows indicate fractures.
